# Comparison of existing polypharmacy measures to a measure incorporating all modes of administration: an observational study using routinely collected data

**DOI:** 10.1007/s11096-026-02194-0

**Published:** 2026-07-31

**Authors:** David Lim, David Youens, Rachael Moorin, Andrew Stafford

**Affiliations:** 1 Curtin School of Diagnostic and Therapeutic Sciences, Curtin University, GPO Box U1987, Perth, WA 6845 Australia; 2 School of Population Health, Faculty of Health Sciences, Curtin University, Perth, WA Australia; 3 Cancer Domain, Faculty of Health Sciences, Curtin Medical Research Institute, Curtin University, Bentley, WA Australia; 4 School of Population & Global Health, The University of Western Australia, Perth, WA Australia; 5 enAble Institute, Curtin University, Perth, WA Australia

**Keywords:** Aged, Drug utilization, Observational study, Polypharmacy, Routes of administration, Nursing homes

## Abstract

**Introduction:**

Polypharmacy is often associated with adverse clinical outcomes and increased medication management complexity. However conventional analyses of medicine burden and polypharmacy using administrative data are limited to oral medicines (i.e. tablets and capsules), potentially underestimating overall medicine exposure. These products may significantly increase the burden of medication management and therefore risk to patients.

**Aim:**

This study compared traditional measures of polypharmacy with a measure incorporating all modes of medicine administration and examined how these differences impacted estimates of polypharmacy prevalence among adults aged > 65 years in Western Australia (WA) from 2012/13 to 2018/19, between two independent cohorts: community-dwelling and residential aged care home populations.

**Method:**

Linked medication dispensing data were used to classify and compare medicines between community-dwelling and residential aged care populations in Western Australia. Conventional medicine capture was extended to include medicines via various modes of administration, including creams, ointments, patches, and eye and ear drops. Medicines were classified into five categories: 0, 1, 2–4, 5–8, 9 + medicines, and counts were compared across care settings to assess changes in observed medicine burden and measures of polypharmacy. Comparative analysis was performed between two methods: Method 1 (conventional medicine capture) and Method 2 (all medicines including non-oral preparations).

**Results:**

Inclusion of medicines via mode of administration increased observed medicine burden in both subpopulations, with a greater relative increase in residential aged care. This approach notably contributed to total medicine counts, revealing previously unrecognised medicine exposure. This resulted in absolute increases in the 5 + medicine category of 4.0% in 2012/13 and 3.4% in 2018/19. Among aged care residents, opioid analgesics previously ranked as the most common medicine class using Method 1, was reclassified as separate oral and patch formulations using Method 2, ranking 4th and 5th. Drugs for dry eyes also emerged as the 7th most common medicine class, with only minor ranking changes observed for the other top medicine classes.

**Conclusion:**

Incorporating all modes of medicine administration into a polypharmacy measure substantially altered estimates of medicine burden and identified previously unrecognised exposure, particularly among residential aged care populations. The impact was greatest in higher polypharmacy categories, indicating that conventional approaches may underestimate medicine use in individuals with more complex therapeutic regimens. These findings highlight the importance of accounting for formulation diversity and support the inclusion of mode-of-administration–based measures to provide a more comprehensive assessment of medication exposure and complexity.

**Supplementary Information:**

The online version contains supplementary material available at https://doi.org/10.1007/s11096-026-02194-0.

## Impact statements

- This study is one of the first to provide estimates of polypharmacy attributed to medicines measured by mode of administration, highlighting the limitations of using conventional polypharmacy measures at different thresholds of polypharmacy, particularly in residential aged care populations.
- These findings support safer and more holistic polypharmacy assessment by highlighting the contribution of all medicine preparations to administration complexity and encourages clinicians and pharmacists to consider all formulations and modes of administration when assessing patient’s level of polypharmacy via medicine classes, and potential subsequent deprescribing decisions.
- This study could inform health system planning and medication-management policy through promoting the use of more comprehensive polypharmacy metrics to improve monitoring and evaluation, especially in aged care.

## Introduction

The simultaneous use of multiple medicines, or polypharmacy, is increasingly prevalent among older adults and is traditionally measured by counting oral medicines [1–3]. Polypharmacy prevalence has been reported to range from 33.9% to over 90% among residents of residential aged care homes (RACHs), substantially higher than observed in community-dwelling (CD) populations [4–6]. However, this wide variation is partly attributable to the absence of a standardised definition of polypharmacy, with thresholds ranging from as few as two to more than ten medicines [7, 8]. This definitional inconsistency limits comparability across studies and obscures accurate estimation of medicine burden in older populations.

Compounding this issue, conventional definitions typically restrict polypharmacy measures to oral medicines, thereby neglecting the significant medication exposure and burden associated with medicines classified by all modes of administration, including topical agents, transdermal patches, infusions, ophthalmic and otic preparations. In addition to oral medicines, topical formulations are frequently prescribed in older populations for conditions such as dry eye, glaucoma, dermatological disorders, and chronic pain [9]. Although these therapies can present substantial administration challenges for older adults [10–13], they are rarely incorporated into routine measures of polypharmacy [12, 13]. As a result, current approaches may systematically underestimate total medicine burden and misrepresent differences in medication exposure across care settings.

These considerations align with broader medication complexity frameworks, which recognise administration requirements alongside medicine counts [14]. Incorporating the mode of administration into polypharmacy measures may therefore provide a more comprehensive representation of medicine burden and patient-centred impact across older populations than conventional medicine counts alone.

### Aim

This study compared traditional measures of polypharmacy with a measure incorporating all modes of medicine administration and examined how these differences impacted estimates of polypharmacy prevalence among adults aged > 65 years in Western Australia (WA) from 2012/13 to 2018/19, between two independent cohorts: community-dwelling and residential aged care home populations.

## Method

This study employed a repeated cross-sectional study design using linked administrative data that captured records of medicine dispensing, hospital admissions, emergency department presentations, and mortality. The data sources consisted of the Medicare Enrolment File, Pharmaceutical Benefits Scheme (PBS), Medicare Benefits Schedule (MBS), National Mortality Records (NMR), WA Emergency Department Data Collection (EDDC), and WA Hospital Morbidity Data Collection (HMDC). Detailed information on data collections can be found in Supplementary File 1. Eligible participants included individuals over the age of 65 years and alive for a minimum of two years within the study period (following the Australian financial year running 01 July—30 June), who had at least one record in either MBS, EDDC, or HMDC, in either the current or preceding year. Two subpopulations were identified for analysis: RACH-dwelling and CD (Supplementary File 2).

In method 1 (conventional medicine capture), medicines of interest included all PBS dispensing within the ascertainment window (1st October to 31st December) of each financial year from 2012/13 to 2018/19 inclusive. This ascertainment window was selected as it required a shorter follow-up duration (6 months), thereby enabling the inclusion of a more representative cohort of individuals residing in RACHs, particularly those approaching the end of life. Standard exclusions were applied [3], including the removal of most topical products, non-therapeutic items, medicines assigned under Anatomical Therapeutic Chemical (ATC) category “V” [3].

An exclusion list (Supplementary File 3) containing ATC codes corresponding to excluded topical and non-therapeutic agents, as well as an additional exclusion list (Supplementary File 4) comprising of unique active ingredient names and item codes, are provided. Paracetamol (ATC: N02BE01) was also excluded due to incomplete capture within the PBS over the study period. However, due to inherent limitations in ATC coding conventions (further explained in the limitations section), some non-oral formulations (including certain topical preparations) may remain captured within broader therapeutic classes (e.g., opioids) and are therefore retained in the analysis.

Unique active ingredients were first identified for all PBS items, with each active ingredient in fixed-dose combination products treated as a distinct entity. For example, the combination tablet Exforge®, containing both amlodipine and valsartan, was split into two distinct active ingredients. Subsequently, each ingredient was then grouped into broader medicine classes as defined according to subsections of the Australian Medicines Handbook (AMH) [15]. This approach aligns with prior research that used national formularies to capture drug classes for measuring polypharmacy and avoids double counting of therapeutic substitutions [16]. For example, atorvastatin and simvastatin were classed together since they are not typically prescribed concurrently. A list of medicine classes with their corresponding unique active ingredients is provided in Supplementary File 5.

In Method 2, the scope of medicines was expanded and reclassified using a mode-of-administration framework. Specifically, this approach incorporated additional medicine types that were excluded under Method 1, alongside medicines already captured. Each medicine class was further categorised according to its mode of administration (e.g., oral, topical, eye, ear, inhaled, and parenteral, including intravenous infusions and injectables). Counts were derived from unique medicine class-mode of administration combinations to enable the identification of distinct formulations where a participant was dispensed multiple forms of the same unique active ingredient. Consequently, multiple formulations of the same active ingredient were counted separately when administered via different modes. For example, sublingual buprenorphine was considered separately to buprenorphine delivered via a transdermal patch. A full list of administration modes is provided in Supplementary File 6.

### Comparison of polypharmacy thresholds between methods

Polypharmacy was measured using medicine class counts grouped into five categories according to the person-level cumulative count of distinct medicine classes dispensed (1, 2–4, 5 + , 5–8 and 9 +). The number of dispensing records at the beginning and end of the study was compared between Method 1 and Method 2 summarised using mean and median. The variance in the distributions measured using standard deviation (SD) or inter quartile range (IQR) as appropriate.

### Describing the most frequently dispensed medicine classes

For the 2018/19 financial year, the top ten medicine classes were described in terms of frequency and percentage for each medicine class relative to (i) the total number of dispensed records, and (ii) the total number of individuals. Changes in ranking position between Method 1 and Method 2 were also reported to illustrate the impact of including all modes of medicine administration.

### Ethics approval

Ethics approval for this study was obtained from Curtin University (Approval No. RD-42–14), the Department of Health Western Australia (Approval No. RGS0000002902), and the Australian Institute of Health and Welfare (Approval No. EO2020/2/1138 ID 2533) under a waiver of consent.

## Results

### Overall population distribution of medicine categories

The study cohort included 268,374 individuals in 2012/13 and 346,789 in 2018/19. Females accounted for the majority, in particular among RACH residents (66.9% female in 2012/13, 65.8% in 2018/19). Among the RACH population over 36% were aged 85 and above at both time points, compared to approximately 10% among the CD population. Detailed population characteristics are presented in Supplementary File 7.

The inclusion of all dosage formulations under Method 2 was associated with modest increases in both the number of medicine classes and the total number of dispensing records, when compared to Method 1. In 2012/13, the application of Method 2 resulted in a 4.0% absolute increase in the proportion of individuals in the 5 + medicine category compared with Method 1, largely attributable to an increase in the 9 + medicine category (3.3%). Similarly, in 2018/19, the 5 + medicine category increased by 3.4%, again mainly due to the 9 + medicine category (2.6%). A detailed summary is provided in Table 1.

**Table 1:** Overall population distribution 2012/13 vs 2018/19

	2012/13 N = 268,374 (%)				2018/19 N = 346,786 (%)			
	Distribution of population by Method 1	Distribution of population by Method 2	Absolute difference between Method 1 and Method 2	P-value^1^	Distribution of population by Method 1	Distribution of population by Method 2	Absolute difference between Method 1 and Method 2	P-value^1^
**Medicine category**				< 0.001				< 0.001
0	34,862 (13.0%)	31,960 (11.9%)	*-2,902 (-1.1%)*		45,936 (13.2%)	42,444 (12.2%)	*-3,492 (-1.0%)*	
1	26,320 (9.8%)	23,847 (8.9%)	*-2,473 (-0.9%)*		35,253 (10.2%)	32,413 (9.3%)	*-2,840 (-0.8%)*	
2–4	92,177 (34.3%)	86,878 (32.4%)	*-5,299 (-2.0%)*		121,026 (34.9%)	115,541 (33.3%)	*-5,485 (-1.6%)*	
**5 + **	115,015 (42.9%)	125,689 (46.8%)	** + *10,674 (*+ *4.0%)***		144,571 (41.7%)	156,388 (45.1%)	** + *11,817 (*+ *3.4%)***	
5–8	79,533 (29.6%)	81,442 (30.4%)	+ *1,909 (*+ *0.7%)*		99,822 (28.8%)	102,602 (29.6%)	+ *2,780 (*+ *0.8%)*	
9 +	35,482 (13.2%)	44,247 (16.4%)	+ *8,765 (*+ *3.3%)*		44,749 (12.9%)	53,786 (15.5%)	+ *9,037 (*+ *2.6%)*	
**Number of medicine classes**				< 0.001				< 0.001
Mean (SD)^2^	4.4 (3.6)	4.9 (3.9)			4.4 (3.6)	4.7 (3.8)		
Median (IQR)^3^	4 (2 – 6)	4 (2 – 7)			4 (2 – 6)	4 (2 – 7)		
**Number of dispensing records**				< 0.001				< 0.001
Mean (SD)^2^	10.0 (9.3)	10.7 (9.9)			9.7 (9.4)	10.3 (9.8)		
Median (IQR)^3^	8 (3 – 14)	8 (3 – 15)			7 (3 – 14)	8 (3 – 15)		

Italics indicate that the value represents the calculated absolute difference between Method 1 and Method 2

Bold indicates the medicine category with the highest absolute difference

1 = P-values were derived from Pearson’s chi^2^ test for categorical variables, and t-test for continuous variables

2 = Standard deviation

3 = Inter quartile range

### Comparison of population distribution by subpopulation

Table 2 compares the absolute difference between Method 1 and Method 2, and subpopulation analyses showed that in both 2012/13 and 2018/19, the largest absolute increases occurred in the 9 + medicine category among residents of RACHs (5.9% in 2012/13 and 5.1% in 2018/19), whereas among CD individuals, the greatest increase was observed in the 5 + medicine category (3.9% in 2012/13 and 3.3% in 2018/19). Detailed results are presented in Table 2.

**Table 2:** Comparison of population distribution by subpopulation 2012/13 vs 2018/19

	2012/13							2018/19						
	Community-Dwelling N = 239,120 (%)			Residential Aged Care Homes N = 29,254 (%)				Community-Dwelling N = 315,483 (%)			Residential Aged Care Homes N = 31,303 (%)			
	Distribution by Method 1	Distribution by Method 2	Absolute difference between Method 1 and Method 2	Distribution by Method 1	Distribution by Method 2	Absolute difference between Method 1 and Method 2	P-value^1^	Distribution by Method 1	Distribution by Method 2	Absolute difference between Method 1 and Method 2	Distribution by Method 1	Distribution by Method 2	Absolute difference between Method 1 and Method 2	P-value^1^
**Medicine category**							< 0.001							< 0.001
0	33,243 (13.9%)	30,546 (12.8%)	*-2,697 (-1.1%)*	1,619 (5.5%)	1,414 (4.8%)	*-205 (-0.7%)*		44,076 (14.0%)	40,759 (12.9%)	*-3,317 (-1.1%)*	1,860 (6.0%)	1,685 (5.4%)	*-175 (-0.6%)*	
1	24,716 (10.3%)	22,457 (9.4%)	*-2,259 (-0.9%)*	1,604 (5.5%)	1,390 (4.8%)	*-214 (-0.7%)*		33,376 (10.6%)	30,800 (9.8%)	*-2,576 (-0.8%)*	1,877 (6.0%)	1,613 (5.2%)	*-264 (-0.8%)*	
2–4	84,278 (35.2%)	79,978 (33.4%)	*-4,300 (-1.8%)*	7,899 (27.0%)	6,900 (23.6%)	*-999 (-3.4%)*		112,224 (35.6%)	107,633 (34.1%)	*-4,591 (-1.5%)*	8,802 (28.3%)	7,908 (25.3%)	*-894 (-2.9%)*	
5 +	96,883 (40.5%)	106,139 (44.4%)	** + *9,256 (*+ *3.9%)***	18,132 (62.0%)	19,550 (66.8%)	+ *1,418 (*+ *4.8%)*		125,807 (39.9%)	136,291 (43.2%)	** + *10,484 (*+ *3.3%)***	18,764 (59.7%)	20,097 (64.2%)	+ *1,333 (*+ *4.3%)*	
5–8	68,789 (28.8%)	71,005 (29.7%)	+ *2,216 (*+ *0.9%)*	10,744 (36.7%)	10,437 (35.7%)	*-307 (-1.0%)*		88,527 (28.1%)	91,570 (29.0%)	+ *3,043 (*+ *1.0%)*	11,295 (36.1%)	11,032 (35.2%)	*-263 (-0.8%)*	
9 +	28,094 (11.7%)	35,134 (14.7%)	+ *7,040 (*+ *2.9%)*	7,388 (25.3%)	9,113 (31.2%)	** + *1,725 (*+ *5.9%)***		37,280 (11.8%)	44,721 (14.2%)	+ *7,441 (*+ *2.4%)*	7,469 (23.6%)	9,065 (29.0%)	** + *1,596 (*+ *5.1%)***	
**Number of medicine classes**							< 0.001							< 0.001
Mean (SD)^2^	4.2 (3.5)	4.6 (3.7)		6.1 (3.9)	6.8 (4.4)			4.2 (3.5)	4.5 (3.7)		6.0 (3.9)	6.5 (4.3)		
Median (IQR)^3^	4 (2 – 6)	4 (2 – 7)		6 (3 – 9)	6 (4 – 9)			4 (2 – 6)	4 (2 – 7)		5 (3 – 8)	6 (3 – 9)		
**Number of dispensing records**							< 0.001							< 0.001
Mean (SD)^2^	9.4 (8.9)	10.0 (9.4)		14.8 (11.3)	16.0 (12.1)			9.3 (9.0)	9.8 (9.4)		14.6 (11.5)	15.6 (12.1)		
Median (IQR)^3^	7 (3 – 13)	8 (3 – 14)		13 (7 – 21)	14 (7 – 22)			7 (3 – 13)	7 (3 – 14)		12 (6 – 20)	13 (7 – 22)		

Italics indicate that the value represents the calculated absolute difference between Method 1 and Method 2

Bold indicates the medicine category with the highest absolute difference

1 = P-values were derived from Pearson’s chi^2^ test for categorical variables, and t-test for continuous variables

2 = Standard deviation

3 = Inter quartile range

### The top ten medicine classes in 2018/19

Table 3 presents a comparison of the ten most frequently dispensed medicine classes in 2018/19 based on the number of dispensed records, classified by the two Methods. Notable differences between the most common medicine classes were identified. In particular, for the RACH residents, drugs for dry eyes was the 7th most common medicine class according to Method 2, yet this class was not present in the Method 1 rankings. Method 2 provided additional insight into opioid analgesics, the most highly ranked medicine class according to Method 1; using Method 2, oral and patch formulations ranked as 4th and 9th, respectively. When ranked by the number of people for whom the medicine classes were dispensed (Table 4), for both subpopulations, the top ten medicine classes remained unchanged for both subpopulations following application of Method 2, although minor shifts in ranking positions were observed.

**Table 3:** The top ten medicine classes in 2018/19 based on the number of dispensed records by Method 1 vs. Method 2

Rank position by no. dispensed records	Community-Dwelling						Residential Aged Care Homes					
	Medicine class by Method 1	No. of dispensed records n = 3,353,704 (%)	Change in ranking position (Method 2 compared with Method 1)	Medicine class by Method 2	Mode of administration	No. of dispensed records n = 3,560,865 (%)	Medicine class by Method 1	No. of dispensed records n = 498,560 (%)	Change in ranking position (Method 2 compared with Method 1)	Medicine class by Method 2	Mode of administration	No. of dispensed records n = 542,344 (%)
**1**	RAAS inhibitors ^1^	474,514 (14.1)	Nil	RAAS inhibitors ^1^	oral	474,514 (13.3)	Opioid analgesics	45,501 (9.0)	**↓ to 4 & 9**	RAAS inhibitors ^1^	oral	42,396 (7.8)
**2**	Statins	389,447 (11.6)	Nil	Statins	oral	389,447 (10.9)	RAAS inhibitors ^1^	42,473 (8.4)	**↑ to 1**	Proton pump inhibitors	oral	38,205 (7.0)
**3**	Proton pump inhibitors	250,496 (7.5)	Nil	Proton pump inhibitors	oral	247,189 (6.9)	Proton pump inhibitors	40,211 (8.0)	**↑ to 2**	Statins	oral	33,800 (6.2)
**4**	Calcium channel blockers	205,410 (6.1)	Nil	Calcium channel blockers	oral	205,410 (5.8)	Statins	33,800 (6.7)	**↑ to 3**	Opioid analgesics	oral	28,715 (5.3)
**5**	Thiazide and related diuretics	132,019 (3.9)	Nil	Thiazide and related diuretics	oral	132,019 (3.7)	Calcium channel blockers	19,344 (3.8)	Nil	Calcium channel blockers	oral	19,344 (3.6)
**6**	Beta-blockers	126,768 (3.8)	Nil	Beta-blockers	oral	126,768 (3.6)	Selective serotonin reuptake inhibitors	17,328 (3.4)	Nil	Selective serotonin reuptake inhibitors	oral	17,257 (3.2)
**7**	Opioid analgesics	119,406 (3.6)	Nil	Opioid analgesics	oral	101,522 (2.9)	Beta-blockers	16,100 (3.2)	**↓ to 8**	Drugs for dry eyes	eyes	16,760 (3.1)
**8**	Biguanide	91,303 (2.7)	Nil	Biguanide	oral	91,303 (2.6)	Benzodiazepines	13,639 (2.7)	**↓ to 10**	Beta-blockers	oral	16,099 (3.0)
**9**	Selective serotonin reuptake inhibitors	79,194 (2.4)	Nil	Selective serotonin reuptake inhibitors	oral	79,146 (2.2)	Osmotic laxatives	12,632 (2.5)	**↓ to 11**	Opioid analgesics	patch	15,780 (2.9)
**10**	NSAIDs ^2^	67,552 (2.0)	**↓ to 11**	Factor Xa inhibitors	oral	67,520 (1.9)	Other antidepressants	11,171 (2.2)	**↓ to 12**	Benzodiazepines	oral	13,582 (2.5)

1 = Renin-angiotensin aldosterone system inhibitors

2 = Nonsteroidal anti-inflammatory drug

**Table 4:** The top ten medicine classes in 2018/19 based on the total number of people dispensed by Method 1 vs. Method 2

Rank position by no. of individuals	Community-Dwelling						Residential Aged Care Homes					
	Medicine class by Method 1	No. of individuals n = 1,334,180 (%)	Change in ranking position (Method 2 compared with Method 1)	Medicine class by Method 2	Mode of administration	No. of individuals n = 1,426,864 (%)	Medicine class by Method 1	No. of individuals n = 186,333 (%)	Change in ranking position (Method 2 compared with Method 1)	Medicine class by Method 2	Mode of administration	No. of individuals n = 202,711 (%)
**1**	RAAS inhibitors ^1^	156,148 (11.7)	Nil	RAAS inhibitors ^1^	oral	155,939 (10.9)	RAAS inhibitors ^1^	13,602 (7.3)	Nil	RAAS inhibitors ^1^	oral	13,578 (6.7)
**2**	Statins	133,994 (10.1)	Nil	Statins	oral	133,994 (9.4)	Proton pump inhibitors	12,951 (7.0)	Nil	Proton pump inhibitors	oral	12,279 (6.1)
**3**	Proton pump inhibitors	90,074 (6.8)	Nil	Proton pump inhibitors	oral	89,051 (6.2)	Statins	11,275 (6.1)	Nil	Statins	oral	11,275 (5.6)
**4**	Calcium channel blockers	68,824 (5.2)	Nil	Calcium channel blockers	oral	68,824 (4.8)	Opioid analgesics	8,567 (4.6)	**↓ to 5**	Beta-blockers	oral	6,854 (3.4)
**5**	Beta-blockers	53,886 (4.1)	Nil	Beta-blockers	oral	53,886 (3.8)	Beta-blockers	6,854 (3.7)	**↑ to 4**	Opioid analgesics	oral	6,423 (3.2)
**6**	Thiazide and related diuretics	47,512 (3.6)	Nil	Thiazide and related diuretics	oral	47,290 (3.3)	Calcium channel blockers	6,364 (3.4)	Nil	Calcium channel blockers	oral	6,364 (3.1)
**7**	Opioid analgesics	38,622 (2.9)	**↓ to 9**	Biguanide	oral	36,686 (2.6)	Selective serotonin reuptake inhibitors	5,515 (3.0)	**↓ to 8**	Loop diuretics	oral	5,497 (2.7)
**8**	Biguanide	36,686 (2.8)	**↑ to 7**	NSAIDs ^2^	oral	35,841 (2.5)	Loop diuretics	5,502 (3.0)	**↑ to 7**	Selective serotonin reuptake inhibitors	oral	5,494 (2.7)
**9**	NSAIDs ^2^	36,149 (2.7)	**↑ to 8**	Opioid analgesics	oral	35,185 (2.5)	Osmotic laxatives	5,395 (2.9)	Nil	Osmotic laxatives	Oral powder (reconstituted)	5,265 (2.6)
**10**	Penicillins	33,348 (2.5)	Nil	Penicillins	oral	33,137 (2.3)	Benzodiazepines	4,895 (2.6)	Nil	Benzodiazepines	oral	4,864 (2.4)

1 = Renin-angiotensin aldosterone system inhibitors

2 = Nonsteroidal anti-inflammatory drug

## Discussion

This study has several strengths, including the use of large, population-level routinely collected data and the explicit differentiation of medicines by mode of administration across both CD and RACH settings. The findings indicated that classifying medicines by their mode of administration to include non-oral preparations, contribute substantially to medicine class counts and administrative burden. This broader inclusion of medicines resulted in an estimated 3.4% to 4.0% additional prevalence of polypharmacy, previously uncaptured in studies using conventional polypharmacy measures [3]. The impact of all modes of administration was greatest among residents of RACHs and in higher polypharmacy categories (5 + and especially 9 + medicines), suggesting that using all modes of administration disproportionately affect estimates of medicine burden in RACH residents.

These results likely reflected the greater clinical complexity and increased use of topical and sensory therapies among RACH residents compared with CD adults. To provide context, RACH residents are classified across thirteen Australian National Aged Care Classification funding classes based on assessments of functional capacity, cognitive status and clinical care needs [17, 18]. National data indicate that approximately 182,000 people were residing in permanent residential aged care in Australia during 2018–19, of whom 60% had high care needs for activities of daily living, 64% had high care needs for cognition and behaviour, and 52% had high care needs for complex health care [19]. In addition, 53% had dementia, 49% had depression, and 87% had at least one diagnosed mental health or behavioural condition [19]. These figures highlight the substantial functional dependency and clinical complexity of the Australian residential aged care population, characteristics that are likely to influence patterns of medicine use and contribute to the higher prevalence of polypharmacy that incorporated all modes of administration observed in this setting.

A key finding was the identification of drugs for dry eyes as one of the most frequently dispensed medicine classes under the expanded classification. This highlighted the substantial use of ophthalmic preparations in RACH residents and demonstrated how commonly used non-oral therapies were overlooked in conventional polypharmacy measures. Similarly, the differentiation of medicine classes by mode of administration provided greater insight into formulation-specific use, as illustrated by the separation of opioid analgesics into oral and transdermal medicine class-mode categories. These findings underscored the importance of recognising formulation diversity when evaluating medicine exposure and provided further evidence that medicine counts alone may not adequately capture the complexity of therapeutic regimens.

The inclusion of all modes of administration also has implications for understanding medication management burden. Many non-oral preparations, including eye drops, topical agents, and transdermal patches, require specific administration techniques, coordination, and often require caregiver support, especially those with visual, physical, or cognitive impairment [9, 11, 20]. As such incorporating mode of administration into polypharmacy measures provided a more patient-centred representation of treatment burden, extending beyond numerical medicine counts to capture aspects of administration complexity. This has relevance in aged care settings, where resource allocation, staffing requirements, and clinical workflows are influenced by medication administration demands.

From a practical perspective, these findings suggested several implications for clinical practice and health system planning. For clinicians and pharmacists, incorporating all modes of administration into routine medication reviews may improve identification of patients with high treatment burden and support more informed deprescribing decisions. For example, a patient prescribed multiple eye drops, topical therapies, and oral medicines may exceed conventional polypharmacy measures when all modes of administration are considered, thereby altering clinical assessment and management. At a systems level, expanded polypharmacy measures could inform the refinement of Australia’s National Aged Care Mandatory Quality Indicator Program (NACMQIP) polypharmacy indicator and monitoring framework in aged care, where current metrics do not capture administration-related burden [21]. This may support more accurate evaluation of medication use and improve alignment between policy measures and clinical practice.

These findings should be interpreted within the context of ongoing debate regarding the definition and clinical utility of polypharmacy. While polypharmacy is often associated with adverse outcomes, it may also represent clinically appropriate and necessary management of multimorbidity in older adults [22, 23]. Consequently, there is debate regarding the clinical utility of defining polypharmacy using arbitrary numeric thresholds alone [24]. Contemporary approaches increasingly distinguish between appropriate and inappropriate polypharmacy, emphasising the need to balance minimisation of unnecessary or harmful medicines with preservation of evidence based, beneficial treatments [23, 25–27]. In this context, the present findings did not suggest that higher medicine counts are inherently problematic; rather, they indicated that conventional polypharmacy measures may have underestimated overall treatment complexity and administration burden, particularly in residential aged care settings. Incorporating mode of administration into polypharmacy assessments may therefore be considered a complementary approach to existing methods for evaluating medication use.

Despite extending traditional measure of polypharmacy, this study remained constrained by several limitations. Medication exposure was inferred from administrative dispensing data and did not include information on clinical indication, prescribed dosage, duration of therapy and other patient medical history such as existing comorbidities at the individual level. As a result, the appropriateness of medicine use could not be assessed. In addition, the data was limited to PBS dispensing claims and excluded medicines that were private prescriptions, and non-prescription medicines such as over-the-counter (OTC), complementary and alternative medicines, which were estimated in a previous study [2].

Within this context, paracetamol was not included in the analysis, representing an additional limitation given its frequent use and potential contribution to overall medicine burden. This exclusion reflects two methodological considerations. First, paracetamol use would be substantially under-ascertained because most supply occurs via over-the-counter purchase and is therefore not captured in our PBS dispensing records. Second, paracetamol was not consistently recorded as a PBS benefit across the study period due to its delisting in 2015/16, resulting in incomplete longitudinal capture. Preliminary analyses indicated that inclusion of paracetamol introduced a temporal data artefact, compromising internal validity of comparisons over time. Consequently, the exclusion of paracetamol and other non-prescription medicines likely led to an underestimation of polypharmacy prevalence. The estimates reported in this study should therefore be interpreted as conservative, with the true prevalence of polypharmacy likely to be higher if OTC and non-prescription medicines were comprehensively captured.

Furthermore, although Method 1 followed conventional inclusion and exclusion criteria [3], the use of the World Health Organization Anatomical Therapeutic Chemical/Defined Daily Dose (ATC/DDD) system presents inherent limitations when comparing medicine exposure by mode of administration [28]. Specifically, ATC codes are not consistently assigned with a single route of administration, and some encompass multiple formulations. For example, buprenorphine (classified under ATC codes N02AE01 and N07BC01) includes preparations administered via sublingual, transdermal, and parenteral routes. Consequently, medicines were included where at least one relevant route met the inclusion criteria for Method 1. This approach may have introduced some underestimation, as reflected in a modest increase in polypharmacy counts after expanding the medicines of interest as per Method 2 in our study. This limitation affected the ability to distinguish administration-specific exposure and represents a limitation of the classification system rather than the analytical approach.

Additionally, the classification approach used in Method 2, which differentiated medicine classes by mode of administration, may have resulted in higher medicine class-mode counts where therapeutically similar formulations were counted separately (e.g., oral versus transdermal opioids, or different formulations of PPIs and NSAIDs). While this distinction was intentional and enabled quantification of the impact of administration-specific contributions to medicine burden, it may also have contributed to an apparent increase in polypharmacy prevalence. Accordingly, some differences between Method 1 and Method 2 reflect classification effects rather than entirely distinct therapeutic exposures.

Although the large population sample size reduces the likelihood that findings are driven by random variation or small-sample bias, the limitations outlined above have implications for the interpretation and generalisability of the findings. As the study was conducted within the Australian healthcare context, the results are most applicable to settings with similar data systems, prescribing practices, and aged care structures. Accordingly, extrapolation to other healthcare systems should therefore be undertaken with consideration of these contextual differences.

Future research could address these limitations by enhancing existing data systems through linkage with prescribing or electronic medical record data to capture more granular treatment information, including prescribed dose, duration, and clinical indication. This would enable more accurate assessment of medicine exposure and facilitate evaluation of the relationship between administration-inclusive polypharmacy measures and clinical outcomes, such as hospitalisation and adverse drug events. Further work is also needed to develop and validate refined polypharmacy metrics that integrate both medicine counts and administration complexity, and to examine their utility in informing clinical decision-making and health policy. Improved understanding and validation of these measures would provide the evidence needed to establish their clinical relevance and support their application in both practice and policy settings.

## Conclusion

This study demonstrated that incorporating all modes of medicine administration into a polypharmacy measure substantially altered estimates of medicine burden and identified previously unrecognised exposure, particularly among residential aged care populations. The effect was most pronounced within higher polypharmacy categories, indicating that conventional approaches may underestimate medicine use in individuals with complex therapeutic regimens. Accounting for medicines by mode of administration increased observed medicine counts and highlighted the contribution of formulation diversity to overall treatment burden. These findings underscore the limitations of relying solely on conventional medicine counts and emphasise the value of incorporating mode of administration into routine polypharmacy assessment. Such an approach provides a more comprehensive representation of medication exposure and supports improved evaluation of medication complexity and informing strategies to optimise medicine use across care settings.

## Supplementary Information

Below is the link to the electronic supplementary material.

Supplementary file1 (DOCX 24 kb)

Supplementary file2 (DOCX 23 kb)

Supplementary file3 (DOCX 27 kb)

Supplementary file4 (DOCX 24 kb)

Supplementary file5 (DOCX 71 kb)

Supplementary file6 (DOCX 40 kb)

Supplementary file7 (DOCX 27 kb)

## Data Availability

Due to ethical or privacy reasons the data that support this study cannot be publicly shared.
