# Supplementary material for: Comparison of existing polypharmacy measures to a measure incorporating all modes of administration: an observational study using routinely collected data
Source: Int J Clin Pharm. 2026 Jul 31;48(5):2158–68. doi: 10.1007/s11096-026-02194-0 (PMC13569558; doi:10.1007/s11096-026-02194-0)
Supplement: Supplementary file 1 [file 11096_2026_2194_MOESM1_ESM.docx]

**Supplementary File 1: Detailed information on dataset collections**

**Datasets**

The Medicare Enrolment File provided records pertaining to people aged 45 years and older in WA registered with Medicare and was linked to the following datasets:

- **Pharmaceutical Benefits Scheme (PBS)** which contains information on all medicines dispensed from community pharmacies except for those funded by the Department of Veterans Affairs. This dataset includes Anatomical Therapeutic Chemical (ATC) codes, quantity of dispensing, date of dispensing and PBS item codes.
- **Medicare Benefits Schedule (MBS)** data includes records for all claims for medical services through Medicare, which is Australia’s universal health insurance scheme. Key variables included item numbers and dates of each service rendered to patients, patient’s postcode.
- **National Mortality Records (NMR)** which provided date of death.
- The **WA Emergency Department Data Collection (EDDC)** provided high-level diagnosis, discharge destination (if not admitted) for all ED attendances in WA.
- The **WA Hospital Morbidity Data Collection (HMDC)** captures all admissions to public and private hospitals in WA and included admission and separation dates, discharge destinations, and principal and co-diagnoses using the International Classification of Diseases (ICD) version 10 Australian Modification.
