# Supplementary material for: Comparison of existing polypharmacy measures to a measure incorporating all modes of administration: an observational study using routinely collected data
Source: Int J Clin Pharm. 2026 Jul 31;48(5):2158–68. doi: 10.1007/s11096-026-02194-0 (PMC13569558; doi:10.1007/s11096-026-02194-0)
Supplement: Supplementary file 2 [file 11096_2026_2194_MOESM2_ESM.docx]

**Supplementary File 2: Determining subpopulations**

- **RACH subpopulation**: Determined from linked data, specifically using variables “source of referral” and “mode of separation” from the HMDC and EDDC data collections, together with MBS items (1, 20, 35, 43, 51, 90001, 90020, 90035, 90043, 90051, 903) which specify services rendered within RACHs, were used to determine RACH residency status. For each financial year of interest, individuals were classified to the RACH subpopulation if they had RACH evidence prior to the start of that financial year and (to allow sufficient follow up) were alive for at least the first six months (1^st^ July – 31^st^ Dec) of the financial year. The six-month alive criterion excluded 7.5% of the RACH subpopulation from our study.
- **CD subpopulation:** All others not classified as RACH residents.
