# Supplementary material for: Comparison of existing polypharmacy measures to a measure incorporating all modes of administration: an observational study using routinely collected data
Source: Int J Clin Pharm. 2026 Jul 31;48(5):2158–68. doi: 10.1007/s11096-026-02194-0 (PMC13569558; doi:10.1007/s11096-026-02194-0)
Supplement: Supplementary file 3 [file 11096_2026_2194_MOESM3_ESM.docx]

**Supplementary File 3: Alphanumeric codes for ATC exclusion**

List of prefixed alphanumeric codes for ATC exclusion that pertain to topical and non-therapeutic agents.

Prefix alphanumeric codes for ATC exclusion

C05

D01A

D02

D03

D04

D05A

D06

D07

D08

D10A

D11

G01

G02B

M02

P03

R01A

S01A

S01B

S01E

S01X

S02

S03

V

Specific ATC level 5 codes that were excluded

G03FB05

G03FB08

N02BE01 (excluded after initial analysis due to data artifact)
