# Supplementary material for: Comparison of existing polypharmacy measures to a measure incorporating all modes of administration: an observational study using routinely collected data
Source: Int J Clin Pharm. 2026 Jul 31;48(5):2158–68. doi: 10.1007/s11096-026-02194-0 (PMC13569558; doi:10.1007/s11096-026-02194-0)
Supplement: Supplementary file 4 [file 11096_2026_2194_MOESM4_ESM.docx]

**Supplementary File 4: Exclusion list of unique active ingredient names or item code pertaining to topical preparations**

| Excluded unique active ingredients | | Excluded item codes |
| --- | --- | --- |
| acetic acid  apraclonidine  balsam  bandage  bath emollient  benzydamine  benzyl benzoate  bimatoprost  brimonidine  brinzolamide  calcipotriol  carbomer  carboxymethylcellulose  chlorhexidine  cicloprox  cinchocaine hydrochloride  coal tar  cromoglycate  dichlorobenzene  dichlorobenzene chlorbutol  diphemanil methylsulphate  dorzolamide  dressing  emollient  fluorometholone  gauze  gel  homatropine  hyaluronate sodium  hydroxyquinoline  hypromellose  ichthammol  imiquimod  ingenol mebutate | ketoconazole  lanolin  latanoprost  lauryl sulfoacetate sodium  levocabastine  lubricating agent  menthol & eucalyptus  methyl salicylate  ofloxacin  pad wound debridement  paraffin  perfluorohexyloctane  permethrin  peru balsam  pine tar  podophyllotoxin  ricinoleic acid  salicylic acid  saliva substitute  silver sulfadiazine  sunscreens  tafluprost  talc  tape  timolol  travatoprost  travoprost  triamcinolone  urea  urine  various  wool alcohols  zinc oxide  zoline | 1093M  193M |
