# Supplementary material for: Comparison of existing polypharmacy measures to a measure incorporating all modes of administration: an observational study using routinely collected data
Source: Int J Clin Pharm. 2026 Jul 31;48(5):2158–68. doi: 10.1007/s11096-026-02194-0 (PMC13569558; doi:10.1007/s11096-026-02194-0)
Supplement: Supplementary file 5 [file 11096_2026_2194_MOESM5_ESM.docx]

**Supplementary File 5: List of medicine classes and corresponding unique active ingredients**

| **Medicine class** | **Unique active ingredient** |
| --- | --- |
| 5-alpha-reductase inhibitors | dutasteride |
| 5-alpha-reductase inhibitors | finasteride |
| 5-aminosalicylates | olsalazine |
| 5-aminosalicylates | balsalazide |
| 5-aminosalicylates | mesalazine |
| 5-aminosalicylates | sulfasalazine |
| 5HT3 antagonists | tropisetron |
| 5HT3 antagonists | granisetron |
| 5HT3 antagonists | palonosetron |
| 5HT3 antagonists | ondansetron |
| alcohol dependence | naltrexone |
| alcohol dependence | acamprosate |
| aldosterone antagonists | eplerenone |
| aldosterone antagonists | spironolactone |
| alkylating agents | bendamustine |
| alkylating agents | ifosfamide |
| alkylating agents | busulfan |
| alkylating agents | carmustine |
| alkylating agents | fotemustine |
| alkylating agents | temozolomide |
| alkylating agents | chlorambucil |
| alkylating agents | melphalan |
| alkylating agents | cyclophosphamide |
| amino acid | lysine |
| amino acid | glycine |
| aminoglycosides | gentamicin |
| aminoglycosides | tobramycin |
| androgens | testosterone |
| antacids | aluminium hydroxide |
| antacids | magnesium trisilicate |
| antacids | sodium alginate |
| antacids | magnesium hydroxide |
| antacids | simethicone |
| anthracyclines | idarubicin |
| anthracyclines | mitozantrone |
| anthracyclines | epirubicin |
| anthracyclines | doxorubicin |
| antiandrogens | nilutamide |
| antiandrogens | flutamide |
| antiandrogens | enzalutamide |
| antiandrogens | cyproterone |
| antiandrogens | bicalutamide |
| antiarrhythmics | disopyramide |
| antiarrhythmics | amiodarone |
| antiarrhythmics | lignocaine |
| antiarrhythmics | sotalol |
| antiarrhythmics | flecainide |
| antiarrhythmics | digoxin |
| antibody-drug conjugates | trastuzumab emtansine |
| antibody-drug conjugates | brentuximab vedotin |
| anticancer antibodies | pembrolizumab |
| anticancer antibodies | ipilimumab |
| anticancer antibodies | cetuximab |
| anticancer antibodies | pertuzumab |
| anticancer antibodies | blinatumomab |
| anticancer antibodies | obinutuzumab |
| anticancer antibodies | bevacizumab |
| anticancer antibodies | rituximab |
| anticancer antibodies | atezolizumab |
| anticancer antibodies | nivolumab |
| anticancer antibodies | trastuzumab |
| anticancer antibodies | durvalumab |
| anticancer antibodies | avelumab |
| anticancer antibodies | panitumumab |
| anticholinergics | benzatropine |
| anticholinergics | atropine |
| anticholinergics | biperiden |
| anticholinergics | atropine sulfate |
| anticholinergics | trihexyphenidyl (benzhexol) |
| anticholinergics (genitourinary) | propantheline |
| anticholinergics (genitourinary) | oxybutynin |
| anticholinergics (inhaled) | aclidinium |
| anticholinergics (inhaled) | umeclidinium |
| anticholinergics (inhaled) | tiotropium |
| anticholinergics (inhaled) | umeclindium |
| anticholinergics (inhaled) | ipratropium |
| anticholinergics (inhaled) | glycopyrronium |
| anticholinesterases | donepezil |
| anticholinesterases | rivastigmine |
| anticholinesterases | galantamine |
| anticholinesterases in myasthenia gravis | pyridostigmine |
| antidote | glucagon hydrochloride |
| antidote | naloxone |
| antifibrinolytic | tranexamic acid |
| antimalarials | atovaquone |
| antimalarials | proguanil |
| antimalarials | artemether |
| antimalarials | quinine |
| antimalarials | lumefantrine |
| antimetabolites | hydroxyurea |
| antimycobacterials | isoniazid |
| antipsychotics | paliperidone |
| antipsychotics | aripiprazole |
| antipsychotics | lurasidone |
| antipsychotics | flupentixol decanoate |
| antipsychotics | trifluoperazine |
| antipsychotics | risperidone |
| antipsychotics | olanzapine |
| antipsychotics | zuclopenthixol decanoate |
| antipsychotics | fluphenazine decanoate |
| antipsychotics | amisulpride |
| antipsychotics | clozapine |
| antipsychotics | ziprasidone |
| antipsychotics | periciazine |
| antipsychotics | chlorpromazine |
| antipsychotics | quetiapine |
| antipsychotics | brexpiprazole |
| antipsychotics | haloperidol |
| antithyroid drugs | carbimazole |
| antithyroid drugs | propylthiouracil |
| antivirals for hepatitis B | peginterferon alfa-2a |
| antivirals for hepatitis B | entecavir |
| antivirals for hepatitis B | adefovir |
| aromatase inhibitors | letrozole |
| aromatase inhibitors | exemestane |
| aromatase inhibitors | anastrozole |
| azoles | posaconazole |
| azoles | voriconazole |
| azoles | itraconazole |
| azoles | fluconazole |
| barbiturates | primidone |
| barbiturates | phenobarbital |
| benzodiazepines | diazepam |
| benzodiazepines | clonazepam |
| benzodiazepines | flunitrazepam |
| benzodiazepines | bromazepam |
| benzodiazepines | temazepam |
| benzodiazepines | alprazolam |
| benzodiazepines | nitrazepam |
| benzodiazepines | oxazepam |
| beta-blockers | oxprenolol |
| beta-blockers | carvedilol |
| beta-blockers | atenolol |
| beta-blockers | metoprolol |
| beta-blockers | labetalol |
| beta-blockers | nebivolol |
| beta-blockers | pindolol |
| beta-blockers | propranolol |
| beta-blockers | bisoprolol |
| biguanide | metformin |
| bisphosphonates | tiludronic acid |
| bisphosphonates | alendronate |
| bisphosphonates | risedronate |
| bisphosphonates | pamidronate disodium |
| bisphosphonates | clodronate |
| bisphosphonates | ibandronate |
| bisphosphonates | risedronic sodium |
| bisphosphonates | zoledronic acid |
| calcineurin inhibitors | tacrolimus |
| calcineurin inhibitors | ciclosporin |
| calcium channel blockers | verapamil |
| calcium channel blockers | diltiazem |
| calcium channel blockers | amlodipine |
| calcium channel blockers | felodipine |
| calcium channel blockers | lercanidipine |
| calcium channel blockers | nifedipine |
| cephalosporins | cefalothin |
| cephalosporins | cefazolin |
| cephalosporins | cefotaxime |
| cephalosporins | ceftriaxone |
| cephalosporins | cefalexin |
| cephalosporins | cefepime |
| cephalosporins | cefaclor |
| cephalosporins | cefuroxime |
| corticosteroids | hydrocortisone |
| corticosteroids | prednisolone oral |
| corticosteroids | betamethasone |
| corticosteroids | prednisolone |
| corticosteroids | fludrocortisone |
| corticosteroids | cortisone |
| corticosteroids | dexamethasone |
| corticosteroids | prednisone |
| corticosteroids | methylprednisolone |
| corticosteroids | triamcinolone |
| corticosteroids | hydrocortisone oral |
| corticosteroids (inhaled) | beclometasone |
| corticosteroids (inhaled) | budesonide |
| corticosteroids (inhaled) | fluticasone propionate |
| corticosteroids (inhaled) | ciclesonide |
| corticosteroids (inhaled) | fluticasone furoate |
| CYP3A inhibitor | cobicistat |
| dipeptidyl peptidase-4 inhibitors | vildagliptin |
| dipeptidyl peptidase-4 inhibitors | linagliptin |
| dipeptidyl peptidase-4 inhibitors | sitagliptin |
| dipeptidyl peptidase-4 inhibitors | alogliptin |
| dipeptidyl peptidase-4 inhibitors | saxagliptin |
| direct thrombin inhibitors | dabigatran |
| direct thrombin inhibitors | bivalirudin |
| dopamine agonists | pramipexole |
| dopamine agonists | rotigotine |
| dopamine agonists | apomorphine |
| dopamine agonists | pergolide |
| dopamine agonists | quinagolide |
| dopamine agonists | bromocriptine |
| dopamine agonists | cabergoline |
| dopamine antagonists | prochlorperazine |
| dopamine antagonists | domperidone |
| dopamine antagonists | metoclopramide |
| drugs affecting GI motility | mebeverine |
| drugs affecting GI motility | hyoscine butylbromide |
| drugs for bipolar disorder | lithium carbonate |
| drugs for endometriosis | danazol |
| drugs for nicotine dependence | bupropion |
| drugs for nicotine dependence | varenicline |
| drugs for nicotine dependence | nicotine |
| drugs for other neurological conditions | riluzole |
| drugs for other neurological conditions | baclofen |
| drugs for other neurological conditions | tetrabenazine |
| drugs for other neurological conditions | dantrolene |
| drugs used in cystic fibrosis | dornase alfa |
| endothelin antagonists | bosentan |
| endothelin antagonists | ambrisentan |
| endothelin antagonists | macitentan |
| erythropoietin agonists | darbepoetin alfa |
| erythropoietin agonists | methoxy polyethylene glycol-epoetin beta |
| erythropoietin agonists | epoetin |
| estrogens | estriol |
| estrogens | ethinylestradiol |
| estrogens | estradiol |
| estrogens | oestradiol |
| factor xa inhibitors | apixaban |
| factor xa inhibitors | rivaroxaban |
| factor xa inhibitors | fondaparinux |
| fibrates | gemfibrozil |
| fibrates | fenofibrate |
| folic acid antagonists | pemetrexed |
| folic acid antagonists | methotrexate |
| folic acid antagonists | raltitrexed |
| folic acid antagonists | pyrimethamine |
| gabapentinoids | pregabalin |
| gabapentinoids | gabapentin |
| G-CSFs | lipegfilgrastim |
| G-CSFs | pegfilgrastim |
| G-CSFs | lenograstim |
| G-CSFs | filgrastim |
| GLP1 analogues | dulaglutide |
| GLP1 analogues | exenatide |
| glycopeptides | vancomycin |
| glycoprotein inhibitors | abciximab |
| glycoprotein inhibitors | eptifibatide |
| glycoprotein inhibitors | tirofiban |
| GnRH antagonists | ganirelix |
| gonadotrophins | choriogonadotropin alfa |
| gonadotrophins | follitropin alfa |
| gonadotrophins | gonadotrophin chorionic human |
| GRH agonists | leuprorelin |
| GRH agonists | triptorelin |
| GRH agonists | goserelin |
| guanine analogues | ganciclovir |
| guanine analogues | famciclovir |
| guanine analogues | valganciclovir |
| guanine analogues | aciclovir |
| guanine analogues | valaciclovir |
| H2 antagonists | ranitidine |
| H2 antagonists | cimetidine |
| H2 antagonists | nizatidine |
| H2 antagonists | famotidine |
| heparins | enoxaparin sodium |
| heparins | dalteparin |
| heparins | heparin |
| hepatitis | interferon alfa-2a |
| hepatitis | interferon alfa-2b |
| hepatitis C | daclatasvir |
| hepatitis C | pibrentasvir |
| hepatitis C | elbasvir |
| hepatitis C | sofosbuvir |
| hepatitis C | ribavirin |
| hepatitis C | ombitasvir |
| hepatitis C | ledipasvir |
| hepatitis C | velpatasvir |
| hepatitis C | dasabuvir |
| hepatitis C | voxilaprevir |
| hepatitis C | boceprevir |
| hepatitis C | paritaprevir |
| hepatitis C | peginterferon alfa-2b |
| hepatitis C | glecaprevir |
| HIV-protease inhibitors | atazanavir |
| HIV-protease inhibitors | lopinavir |
| HIV-protease inhibitors | grazoprevir |
| HIV-protease inhibitors | darunavir |
| HIV-protease inhibitors | ritonavir |
| hydralazine | hydralazine hydrochloride |
| hydralazine | hydralazine |
| immunosuppressants (dermatology) | tildrakizumab |
| immunosuppressants (dermatology) | guselkumab |
| immunosuppressants (dermatology) | risankizumab |
| immunosuppressants (dermatology) | secukinumab |
| immunosuppressants (dermatology) | ustekinumab |
| immunosuppressants (dermatology) | ixekizumab |
| insulin | insulin aspart protamine |
| insulin | insulin degludec |
| insulin | insulin aspart |
| insulin | insulin lispro protamine |
| insulin | insulin glargine |
| insulin | insulin lispro |
| insulin | insulin glulisine |
| insulin | insulin detemir |
| insulin | insulin isophane human |
| insulin | insulin (beef) |
| insulin | insulin neutral human |
| integrase inhibitors | elvitegravir |
| integrase inhibitors | raltegravir |
| integrase inhibitors | bictegravir |
| integrase inhibitors | dolutegravir |
| iron | iron oral |
| iron | ferric pyrophosphate |
| iron | iron parenteral |
| janus kinase inhibitors | upadacitinib |
| janus kinase inhibitors | tofacitinib |
| janus kinase inhibitors | baricitinib |
| kinase inhibitors | abemaciclib |
| kinase inhibitors | lenvatinib |
| kinase inhibitors | lapatinib |
| kinase inhibitors | axitinib |
| kinase inhibitors | dabrafenib |
| kinase inhibitors | ruxolitinib |
| kinase inhibitors | ponatinib |
| kinase inhibitors | binimetinib |
| kinase inhibitors | pazopanib |
| kinase inhibitors | ribociclib |
| kinase inhibitors | palbociclib |
| kinase inhibitors | sorafenib |
| kinase inhibitors | cabozantinib |
| kinase inhibitors | alectinib |
| kinase inhibitors | sunitinib |
| kinase inhibitors | nilotinib |
| kinase inhibitors | nintedanib |
| kinase inhibitors | encorafenib |
| kinase inhibitors | afatinib |
| kinase inhibitors | osimertinib |
| kinase inhibitors | vemurafenib |
| kinase inhibitors | gefitinib |
| kinase inhibitors | imatinib |
| kinase inhibitors | crizotinib |
| kinase inhibitors | ceritinib |
| kinase inhibitors | dasatinib |
| kinase inhibitors | cobimetinib |
| kinase inhibitors | idelalisib |
| kinase inhibitors | trametinib |
| kinase inhibitors | ibrutinib |
| kinase inhibitors | erlotinib |
| less sedating antihistamines | loratadine |
| less sedating antihistamines | cetirizine |
| less sedating antihistamines | fexofenadine |
| lincosamides | lincomycin |
| lincosamides | clindamycin |
| long acting beta2 agonists | salmeterol |
| long acting beta2 agonists | olodaterol |
| long acting beta2 agonists | vilanterol |
| long acting beta2 agonists | formoterol |
| long acting beta2 agonists | indacaterol |
| loop diuretics | etacrynic acid |
| loop diuretics | furosemide |
| macrolides | azithromycin |
| macrolides | clarithromycin |
| macrolides | roxithromycin |
| macrolides | erythromycin |
| MAOIs | tranylcypromine |
| MAOIs | phenelzine |
| mast cell stabiliser | nedocromil |
| mast cell stabiliser | cromoglicic acid |
| miscellaneous | r05x |
| miscellaneous | z |
| miscellaneous | medicinea14 |
| miscellaneous | g02c |
| miscellaneous | medicineb06 |
| miscellaneous | medicined02 |
| miscellaneous | kaolin |
| miscellaneous | spirit |
| miscellaneous | rhamnus frangula |
| miscellaneous | medicinea16 |
| miscellaneous | medicinep02 |
| miscellaneous | medicinea05 |
| miscellaneous ophthalmic agents | verteporfin |
| monoamine oxidase type B inhibitors | selegiline |
| monoamine oxidase type B inhibitors | safinamide |
| monoamine oxidase type B inhibitors | rasagiline |
| mTOR inhibitors | sirolimus |
| mTOR inhibitors | everolimus |
| nitrates | isosorbide mononitrate |
| nitrates | isosorbide dinitrate |
| nitrates | glyceryl trinitrate |
| NNRTIs | rilpivirine |
| NNRTIs | efavirenz |
| NNRTIs | etravirine |
| NNRTIs | nevirapine |
| non-amphetamine psychostimulants | armodafinil |
| non-amphetamine psychostimulants | modafinil |
| non-opioid analgesics | paracetamol |
| nonselective alpha-blockers | phenoxybenzamine |
| NRTIs | zidovudine |
| NRTIs | emtricitabine |
| NRTIs | abacavir |
| NRTIs | lamivudine |
| NSAIDs | mefenamic acid |
| NSAIDs | ibuprofen |
| NSAIDs | naproxen |
| NSAIDs | meloxicam |
| NSAIDs | celecoxib |
| NSAIDs | ketoprofen |
| NSAIDs | tiaprofenic acid |
| NSAIDs | piroxicam |
| NSAIDs | diclofenac |
| NSAIDs | indometacin |
| opioid analgesics | morphine |
| opioid analgesics | buprenorphine |
| opioid analgesics | methadone |
| opioid analgesics | tramadol |
| opioid analgesics | hydromorphone |
| opioid analgesics | opium |
| opioid analgesics | oxycodone |
| opioid analgesics | fentanyl |
| opioid analgesics | codeine |
| opioid analgesics | tapentadol |
| opioid antidiarrhoeals | diphenoxylate |
| opioid antidiarrhoeals | loperamide |
| opioid antitussive | pholcodine |
| oral decongestants | pseudoephedrine |
| oral decongestants | senega ammonia |
| oral minerals and electrolytes and bloods | potassium |
| oral minerals and electrolytes and bloods | sodium chloride |
| oral minerals and electrolytes and bloods | magnesium |
| oral minerals and electrolytes and bloods | hydroxyethyl starch |
| oral minerals and electrolytes and bloods | sodium lactate |
| oral minerals and electrolytes and bloods | sodium bicarbonate |
| oral minerals and electrolytes and bloods | glucose |
| oral minerals and electrolytes and bloods | folic acid |
| oral minerals and electrolytes and bloods | calcium |
| osmotic laxatives | macrogol-3350 |
| osmotic laxatives | lactulose |
| osmotic laxatives | sorbitol |
| osmotic laxatives | glycerol |
| other antianginal drugs | nicorandil |
| other antianginal drugs | perhexiline |
| other antianginal drugs | ivabradine |
| other antibacterials | nitrofurantoin |
| other antibacterials | tinidazole |
| other antibacterials | metronidazole |
| other antibacterials | sulfamethoxazole |
| other antibacterials | trimethoprim |
| other antibacterials | fusidate |
| other antibacterials | methenamine hippurate |
| other anticancer drugs | olaparib |
| other anticancer drugs | etoposide |
| other anticancer drugs | venetoclax |
| other anticancer drugs | eribulin |
| other anticancer drugs | mycobacterium bovis bcg tice strain |
| other anticancer drugs | sonidegib |
| other anticancer drugs | bleomycin |
| other anticancer drugs | vismodegib |
| other anticancer drugs | vorinostat |
| other anticancer drugs | arsenic |
| other antidepressants | moclobemide |
| other antidepressants | mianserin |
| other antidepressants | reboxetine |
| other antidepressants | mirtazapine |
| other antiepileptics | vigabatrin |
| other antiepileptics | tiagabine |
| other antiepileptics | ethosuximide |
| other antiepileptics | brivaracetam |
| other antiepileptics | sulthiame |
| other antiepileptics | oxcarbazepine |
| other antiepileptics | levetiracetam |
| other antiepileptics | zonisamide |
| other antiepileptics | lacosamide |
| other antiepileptics | perampanel |
| other antiepileptics | phenytoin |
| other antiepileptics | valproate |
| other antiepileptics | carbamazepine |
| other antiepileptics | lamotrigine |
| other antiepileptics | topiramate |
| other antifungals | terbinafine |
| other antifungals | nystatin |
| other antifungals | amphotericin b |
| other antifungals | griseofulvin |
| other antihypertensives | prazosin |
| other antihypertensives | clonidine |
| other antihypertensives | moxonidine |
| other antihypertensives | minoxidil |
| other antihypertensives | methyldopa |
| other anti-platelet drugs | aspirin |
| other anti-platelet drugs | dipyridamole |
| other drugs affecting bone | denosumab |
| other drugs affecting bone | cinacalcet |
| other drugs affecting bone | raloxifene |
| other drugs affecting bone | calcitonin salmon |
| other drugs affecting bone | teriparatide |
| other drugs affecting bone | strontium |
| other drugs for ADHD | atomoxetine |
| other drugs for alzheimers disease | memantine |
| other drugs for anxiety and sleep disorders | zopiclone |
| other drugs for anxiety and sleep disorders | buspirone |
| other drugs for asthma | mepolizumab |
| other drugs for asthma | benralizumab |
| other drugs for asthma | omalizumab |
| other drugs for asthma | montelukast |
| other drugs for diabetes | acarbose |
| other drugs for dyslipidaemia | ezetimibe |
| other drugs for dyslipidaemia | colestyramine |
| other drugs for dyslipidaemia | colestipol hydrochloride |
| other drugs for gout | probenecid |
| other drugs for gout | colchicine |
| other drugs for heart failure | sacubitril |
| other drugs for IBD | vedolizumab |
| other drugs for infertility | asenapine |
| other drugs for infertility | clomifene |
| other drugs for MS | ocrelizumab |
| other drugs for MS | interferon beta-1a |
| other drugs for MS | ofatumumab |
| other drugs for MS | natalizumab |
| other drugs for MS | glatiramer acetate |
| other drugs for MS | interferon beta-1b |
| other drugs for MS | daclizumab |
| other drugs for MS | peginterferon beta-1a |
| other drugs for MS | dimethyl fumarate |
| other drugs for MS | teriflunomide |
| other drugs for parkinsons disease | entacapone |
| other drugs for parkinsons disease | levodopa decarboxylase inhibitor |
| other drugs for parkinsons disease | amantadine |
| other drugs for pulmonary hypertension | riociguat |
| other drugs for sexual dysfunction | alprostadil |
| other drugs for ulcers | sucralfate |
| other drugs for urinary incontinence | desmopressin |
| other drugs to prevent migraine | pizotifen |
| other drugs to prevent migraine | methysergide |
| other drugs used with anticancer drugs | plerixafor |
| other gastrointestinal drugs | pancreatic extract |
| other gastrointestinal drugs | orlistat |
| other HIV-antiretroviral | didanosine |
| other HIV-antiretroviral | j05ar |
| other HIV-antiretroviral | stavudine |
| other hormonal anticancer drugs | degarelix |
| other hormonal anticancer drugs | abiraterone |
| other immunomodulators | tocilizumab |
| other immunomodulators | anakinra |
| other immunomodulators | auranofin |
| other immunomodulators | abatacept |
| other immunomodulators | mycophenolate |
| other immunomodulators | hydroxychloroquine |
| other immunomodulators | sodium aurothiomalate |
| other immunomodulators | azathioprine |
| other immunomodulators | leflunomide |
| other laxatives | frangula bark |
| other laxatives | psyllium husk powder |
| other laxatives | citric acid |
| other laxatives | methylnaltrexone |
| other laxatives | sterculia |
| other respiratory drugs | pirfenidone |
| p2y12 antagonists | prasugrel |
| p2y12 antagonists | ticlopidine hydrochloride |
| p2y12 antagonists | ticagrelor |
| p2y12 antagonists | clopidogrel |
| PCSK9 inhibitors | evolocumab |
| penicillins | phenoxymethylpenicillin |
| penicillins | amoxycillin |
| penicillins | ticarcillin-clavulanic acid |
| penicillins | penicillamine |
| penicillins | procaine benzylpenicillin (procaine penicillin) |
| penicillins | benzylpenicillin |
| penicillins | amoxicillin |
| penicillins | ampicillin |
| penicillins | benzathine benzylpenicillin |
| penicillins | dicloxacillin |
| penicillins | amoxicillin clavulanic acid |
| penicillins | flucloxacillin |
| phosphodiesterase 5 inhibitors | tadalafil |
| phosphodiesterase 5 inhibitors | vardenafil |
| phosphodiesterase 5 inhibitors | sildenafil |
| phosphodiesterase 5 inhibitors | avanafil |
| platinum compounds | cisplatin |
| platinum compounds | carboplatin |
| platinum compounds | oxaliplatin |
| progesterone receptor antagonists | mifepristone |
| progestogens | norethisterone |
| progestogens | etonogestrel |
| progestogens | megestrol |
| progestogens | progesterone |
| progestogens | levonorgestrel |
| progestogens | medroxyprogesterone |
| progestogens | norethisterone acetate |
| progestogens | dydrogesterone |
| prostacyclines | iloprost |
| prostaglandins | misoprostol |
| proteasome inhibitors | carfilzomib |
| proteasome inhibitors | bortezomib |
| proton pump inhibitors | pantoprazole |
| proton pump inhibitors | omeprazole |
| proton pump inhibitors | lansoprazole |
| proton pump inhibitors | esomeprazole |
| proton pump inhibitors | rabeprazole |
| psychostimulants | lisdexamfetamine |
| psychostimulants | dexamfetamine |
| psychostimulants | methylphenidate |
| purine antagonists | tioguanine |
| purine antagonists | cladribine |
| purine antagonists | fludarabine |
| pyrimidine antagonists | tipiracil |
| pyrimidine antagonists | gemcitabine |
| pyrimidine antagonists | cytarabine |
| pyrimidine antagonists | fluorouracil |
| pyrimidine antagonists | trifluridine |
| pyrimidine antagonists | azacitidine |
| pyrimidine antagonists | capecitabine |
| quinolones | ciprofloxacin |
| quinolones | norfloxacin |
| RAAS inhibitors | fosinopril |
| RAAS inhibitors | irbesartan |
| RAAS inhibitors | trandolapril |
| RAAS inhibitors | enalapril |
| RAAS inhibitors | lisinopril |
| RAAS inhibitors | losartan |
| RAAS inhibitors | perindopril |
| RAAS inhibitors | captopril |
| RAAS inhibitors | telmisartan |
| RAAS inhibitors | candesartan |
| RAAS inhibitors | eprosartan |
| RAAS inhibitors | quinapril |
| RAAS inhibitors | olmesartan |
| RAAS inhibitors | ramipril |
| RAAS inhibitors | valsartan |
| retinoids | isotretinoin |
| retinoids | acitretin |
| rifamycins | rifampicin |
| rifamycins | rifabutin |
| rifamycins | rifaximin |
| sedating antihitamines | cyproheptadine |
| sedating antihitamines | promethazine |
| selective alpha-blockers | alfuzosin |
| selective alpha-blockers | tamsulosin |
| selective alpha-blockers | terazosin |
| selective estrogen receptor modulators | tamoxifen |
| selective estrogen receptor modulators | toremifene |
| selective serotonin reuptake inhibitors | fluvoxamine |
| selective serotonin reuptake inhibitors | citalopram |
| selective serotonin reuptake inhibitors | escitalopram |
| selective serotonin reuptake inhibitors | paroxetine |
| selective serotonin reuptake inhibitors | fluoxetine |
| selective serotonin reuptake inhibitors | sertraline |
| serotonin and noradrenaline reuptake inhibitors | venlafaxine |
| serotonin and noradrenaline reuptake inhibitors | desvenlafaxine |
| serotonin and noradrenaline reuptake inhibitors | duloxetine |
| SGLT2 inhibitors | canagliflozin |
| SGLT2 inhibitors | dapagliflozin |
| SGLT2 inhibitors | empagliflozin |
| SGLT2 inhibitors | ertugliflozin |
| short acting beta2 agonists | terbutaline |
| short acting beta2 agonists | salbutamol |
| somatostatin analogues | octreotide |
| somatostatin analogues | lanreotide |
| specialised drugs | botulinum toxin |
| specialised drugs | bethanechol |
| specialised drugs | tolvaptan |
| specialised drugs | pralatrexate |
| specialised drugs | tetracosactide |
| specialised drugs | eculizumab |
| specialised drugs | pegvisomant |
| specialised drugs | eltrombopag |
| specialised drugs | ranibizumab |
| specialised drugs | aflibercept |
| specialised drugs | romiplostim |
| sphingosine 1-phosphate receptor modulator | fingolimod |
| statins | rosuvastatin |
| statins | simvastatin |
| statins | pravastatin |
| statins | atorvastatin |
| statins | fluvastatin |
| stimulant laxatives | bisacodyl |
| stimulant laxatives | senna |
| stool softeners | docusate |
| stool softeners | lauryl sulfoacetate sodium |
| subtance P antagonists | netupitant |
| subtance P antagonists | fosaprepitant |
| sulfonylureas | gliclazide |
| sulfonylureas | glipizide |
| sulfonylureas | glibenclamide |
| sulfonylureas | glimepiride |
| sympathomimetics | adrenaline (epinephrine) |
| taxanes | docetaxel |
| taxanes | cabazitaxel |
| taxanes | paclitaxel |
| tenofovir | tenofovir alafenamide |
| tenofovir | tenofovir disoproxil |
| tenofovir | tenofovir |
| tetracyclines | minocycline |
| tetracyclines | doxycycline |
| thalidomide analogues | pomalidomide |
| thalidomide analogues | lenalidomide |
| thalidomide analogues | thalidomide |
| theophylline | theophylline |
| thiazide and related diuretics | chlortalidone |
| thiazide and related diuretics | indapamide |
| thiazide and related diuretics | amiloride |
| thiazide and related diuretics | hydrochlorothiazide |
| thiazolidinediones | rosiglitazone |
| thiazolidinediones | pioglitazone |
| thyroid hormones | liothyronine |
| thyroid hormones | levothyroxine |
| thyroid hormones | thyrotropin alfa |
| tissue plasminogen activator | tenecteplase |
| tissue plasminogen activator | reteplase |
| TNF-alpha antagonists | infliximab |
| TNF-alpha antagonists | certolizumab pegol |
| TNF-alpha antagonists | etanercept |
| TNF-alpha antagonists | adalimumab |
| TNF-alpha antagonists | golimumab |
| topoisomerase I inhibitors | topotecan |
| topoisomerase I inhibitors | irinotecan |
| tricyclic antidepressants | nortriptyline |
| tricyclic antidepressants | amitriptyline |
| tricyclic antidepressants | doxepin |
| tricyclic antidepressants | clomipramine |
| tricyclic antidepressants | dosulepin (dothiepin) |
| tricyclic antidepressants | imipramine |
| triptans | eletriptan |
| triptans | zolmitriptan |
| triptans | sumatriptan |
| triptans | rizatriptan |
| triptans | naratriptan |
| vaccinations | pneumococcal vaccine |
| vaccinations | diphtheria |
| vaccinations | tetanus vaccine |
| vinca alkaloids | vincristine |
| vinca alkaloids | vinblastine |
| vinca alkaloids | vinorelbine |
| vitamin B | hydroxocobalamin |
| vitamin B | thiamine |
| vitamin B | cyanocobalamin |
| vitamin B | pyridoxine |
| vitamin D | calcitriol |
| vitamin D | colecalciferol |
| vitamin K antagonist | warfarin |
| xanthine oxidase inhibitors | allopurinol |
| xanthine oxidase inhibitors | febuxostat |
