# Supplementary material for: Comparison of existing polypharmacy measures to a measure incorporating all modes of administration: an observational study using routinely collected data
Source: Int J Clin Pharm. 2026 Jul 31;48(5):2158–68. doi: 10.1007/s11096-026-02194-0 (PMC13569558; doi:10.1007/s11096-026-02194-0)
Supplement: Supplementary file 6 [file 11096_2026_2194_MOESM6_ESM.docx]

**Supplementary File 6: Modes of administration**

Mode of Administration (description)

Intrauterine device (IUD)

Ear (Ear ointments, ear drops)

Enema

Eye (Eye drops, eye ointments, eye gels)

Head (Shampoo, conditioner, scalp treatments)

Implant

Intravenous infusion

Inhaler (Puffers, inhalants, nebulised)

Injection

Lingual (Sublingual spray, wafer, orally disintegrating tablet, lozenge)

Miscellaneous

Oral (Tablet, capsule, granules consumed by swallowing)

Oral powder (Nutritional formula, oral powder, macrogol sachet)

Mouthwash

Nails (Daktarin tincture, Duofilm)

Nose (Nasal spray, nasal ointment)

Oral liquid (Ready to drink, solution, suspension, elixir)

Patch

Percutaneous endoscopic gastrostomy (PEG)

Skin (Cream, ointment, paste, lotion, liniment, antiseptic)

Spray (Spray to the skin)

Suppository

Vaginal (Pessaries, Ovestin)
