# Supplementary material for: Comparison of existing polypharmacy measures to a measure incorporating all modes of administration: an observational study using routinely collected data
Source: Int J Clin Pharm. 2026 Jul 31;48(5):2158–68. doi: 10.1007/s11096-026-02194-0 (PMC13569558; doi:10.1007/s11096-026-02194-0)
Supplement: Supplementary file 7 [file 11096_2026_2194_MOESM7_ESM.docx]

**Supplementary File 7: Overall Population Characteristics**

|  | **2012/13** | | |  | **2018/19** | | |
| --- | --- | --- | --- | --- | --- | --- | --- |
|  | **Total** | **Community-Dwelling** | **Residential**  **Aged Care Homes** |  | **Total** | **Community-Dwelling** | **Residential**  **Aged Care Homes** |
|  | N=268,374 | N=239,120 | N=29,254 |  | N=346,786 | N=315,483 | N=31,303 |
| **Sex** |  |  |  |  |  |  |  |
| Females | 143,746 (53.6%) | 124,176 (51.9%) | 19,570 (66.9%) |  | 183,394 (52.9%) | 162,789 (51.6%) | 20,605 (65.8%) |
| Males | 124,628 (46.4%) | 114,944 (48.1%) | 9,684 (33.1%) |  | 163,392 (47.1%) | 152,694 (48.4%) | 10,698 (34.2%) |
| **Age** |  |  |  |  |  |  |  |
| >65-74 | 143,545 (53.5%) | 135,490 (56.7%) | 8,055 (27.5%) |  | 191,382 (55.2%) | 181,924 (57.7%) | 9,458 (30.2%) |
| 75-84 | 90,358 (33.7%) | 79,724 (33.3%) | 10,634 (36.4%) |  | 111,984 (32.3%) | 101,710 (32.2%) | 10,274 (32.8%) |
| 85+ | 34,471 (12.8%) | 23,906 (10.0%) | 10,565 (36.1%) |  | 43,420 (12.5%) | 31,849 (10.1%) | 11,571 (37.0%) |
